# Trends in enhanced recovery after surgery (ERAS) in thoracic surgery from a bibliometric insight

**DOI:** 10.1186/s41065-025-00501-9

**Published:** 2025-07-14

**Authors:** Yafeng Liu, Tianze Zhang, Tong Gao, Guanghua Li, Guangquan Xu

**Affiliations:** Department of Thoracic Surgery, Second Affiliated Hospital of Harbin Medical University, Harbin, China

**Keywords:** ERAS, Enhanced recovery after surgery, Thoracic surgery, Bibliometric analysis, VOSviewer, Citespace

## Abstract

**Background:**

In thoracic surgery, the concept of Enhanced Recovery After Surgery (ERAS) has been extensively implemented. Although numerous studies have investigated ERAS in thoracic surgery, bibliometric analyses remain limited. In this study, the developmental trajectory, current research status, and prospective trends of ERAS in thoracic surgery were systematically analyzed through bibliometric and visual analysis techniques.

**Methods:**

Literature pertaining to ERAS in thoracic surgery was retrieved from the Web of Science Core Collection (WoSCC). Microsoft Excel 2019, R software (version 4.1.0), the Bibliometric Online Analysis Platform, VOSviewer (version 1.6.18), and Citespace (version 6.3.R1) were utilized for statistical analysis, bibliometric evaluation, and data visualization.

**Results:**

A total of 617 publications were retrieved over the past decade. The number of publications has generally demonstrated an upward trend from 2015 to 2024. China and Sichuan University ranked as the leading country and institution, respectively, in terms of publication volume. The Journal of Thoracic Disease was identified as the leading journal in both publication count and citation frequency. Henrik Kehlet was recognized as the most prolific and highly co-cited author. Current research hotspots include “video-assisted thoracic surgery,” “pain management,” and “multicenter clinical trials.”

**Conclusion:**

ERAS-related research in thoracic surgery has been increasing steadily, highlighting it as a rapidly evolving and promising field. The ERAS concept plays a critical role in all perioperative phases—preoperative, intraoperative, and postoperative—and requires further in-depth investigation. Many existing ERAS studies in thoracic surgery lack high-quality evidence, underscoring the urgent need for rigorously designed research with robust methodological standards.

## Introduction

The concept of Enhanced Recovery After Surgery (ERAS) was first introduced by Henrik Kehlet in 1997 and initially applied to colorectal surgery [1]. Since the publication of the ERAS consensus on colonic surgery in 2005 [2], ERAS has progressively evolved into a comprehensive perioperative management model, grounded in multidisciplinary collaboration and incorporating various approaches to reduce complications and facilitate accelerated patient recovery [3]. Key areas of thoracic surgery include pulmonary and esophageal surgery, which focus on the diagnosis and treatment of lesions affecting the organs within the thoracic cavity, such as the lungs, esophagus, mediastinum, trachea, chest wall, and pleura. Over the past few decades, thoracic surgery theory and technology have advanced rapidly. The introduction of video-assisted thoracic surgery has significantly transformed thoracic surgery, marking the onset of a minimally invasive era that contrasts sharply with the earlier practice of open thoracotomy [4]. While incision reduction alone is insufficient to meet the growing demands for faster recovery in thoracic surgery patients, due to progressively higher standards for rehabilitation quality, exploring additional strategies to accelerate recovery has become imperative. The development of the ERAS concept is intrinsically linked to the increasing demand for high-quality rehabilitation in thoracic surgery patients and has been applied throughout all perioperative phases, gaining widespread global adoption [5]. Furthermore, the successful application of ERAS in gynecology, urology, and colorectal surgery has demonstrated its viability within the broader surgical field, including thoracic surgery [6–8]. Despite substantial research on the application of ERAS concepts in thoracic surgery, there remains a limited understanding of how ERAS research is evolving within this field. To provide a comprehensive understanding of the history, trends, frontiers, and future directions of the ERAS concept in thoracic surgery, this study performs a bibliometric analysis, summary, and visualization of ERAS-related research over the past decade.

## Materials and methods

### Database and search strategy

**Fig. 1:**
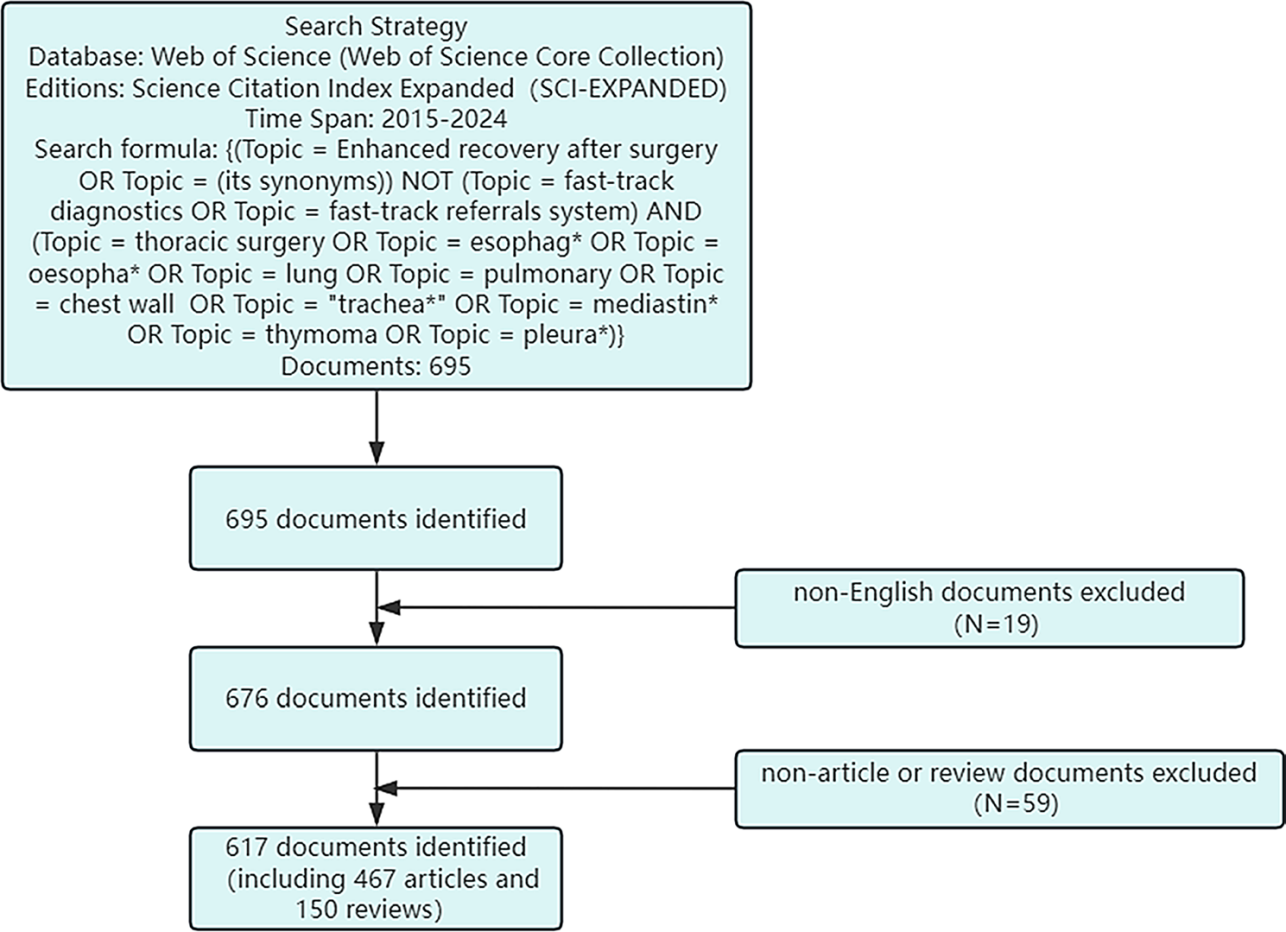
Retrieval strategy and data filtering procedure

A comprehensive search for ERAS-related studies in thoracic surgery was conducted through the Web of Science Core Collection (WoSCC). The search strategy was defined as follows: (Topic = “Enhanced Recovery After Surgery” OR synonyms) NOT (Topic = “fast-track diagnostics” OR “fast-track referral system”) AND (Topic = “thoracic surgery” OR “esophag*” OR “oesopha*” OR “lung” OR “pulmonary” OR “chest wall” OR “trachea*” OR “mediastin*” OR “thymoma” OR “pleura*”). The publication timeframe was restricted to 2015–2024. Only English-language documents classified as articles or reviews were included, while other document types were excluded. Following the initial retrieval of records from the Web of Science Core Collection, a rigorous screening process was conducted to ensure the accuracy and relevance of the dataset. All retrieved publications were manually reviewed to confirm their alignment with the research scope and to identify and eliminate duplicates. In addition to manual screening, automated deduplication was performed using CiteSpace software to further ensure data integrity. To enhance the objectivity and reliability of the process, two authors independently conducted the screening. Any discrepancies were resolved through discussion or by consulting a third author when necessary. Importantly, no filters were applied based on citation frequency, journal impact factor, or any other bibliometric indicator at any stage of the search or screening process. This strategy was deliberately adopted to maintain comprehensiveness and to avoid potential bias toward highly cited literature. Figure 1 illustrates the detailed search strategy and data filtration process.

### Data analysis and visualization

Complete records and cited sources for all papers retrieved from WoSCC were obtained. For data extraction and statistical analysis, the bibliometrix package [9] of R software (version 4.1.0) and Microsoft Excel 2019 were utilized. The Bibliometric Online Analysis Platform (https://bibliometric.com/) was employed to visualize country/region collaboration. Co-authorship analysis of countries/regions, institutions, and authors, as well as co-citation analysis of cited authors, was conducted using VOSviewer (version 1.6.18) [10]. Duplicate detection, co-occurrence analysis of references, cluster and co-occurrence analysis, bursts detection for keywords, and dual-map overlap visualization of journals were performed using Citespace (version 6.3.R1) [11].

## Results

### Publication trend and general characteristics

**Fig. 2:**
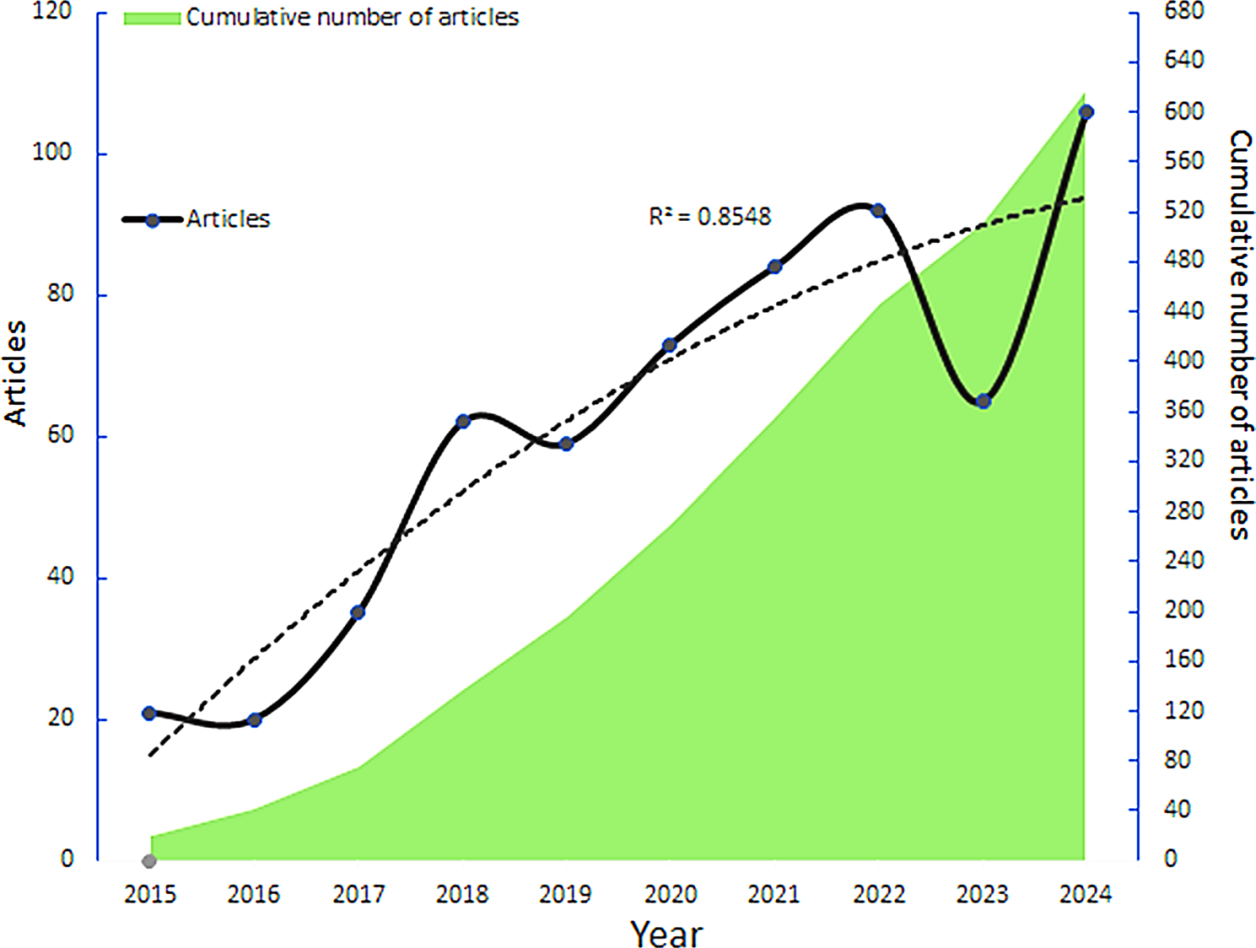
The annual volume and trend of publications on the ERAS in thoracic surgery

Following the search strategy, a total of 467 articles and 150 reviews, totaling 617 papers, were included. Figure 2 illustrates the trend in the annual number of ERAS-related publications in the field of thoracic surgery over the past decade, with over 100 publications projected for 2024. As demonstrated by the fitted curve in Fig. 2 (R²=0.8548), future research on the application of ERAS in thoracic surgery is expected to maintain a high volume.

### Analysis of countries/regions and institutions

**Fig. 3:**
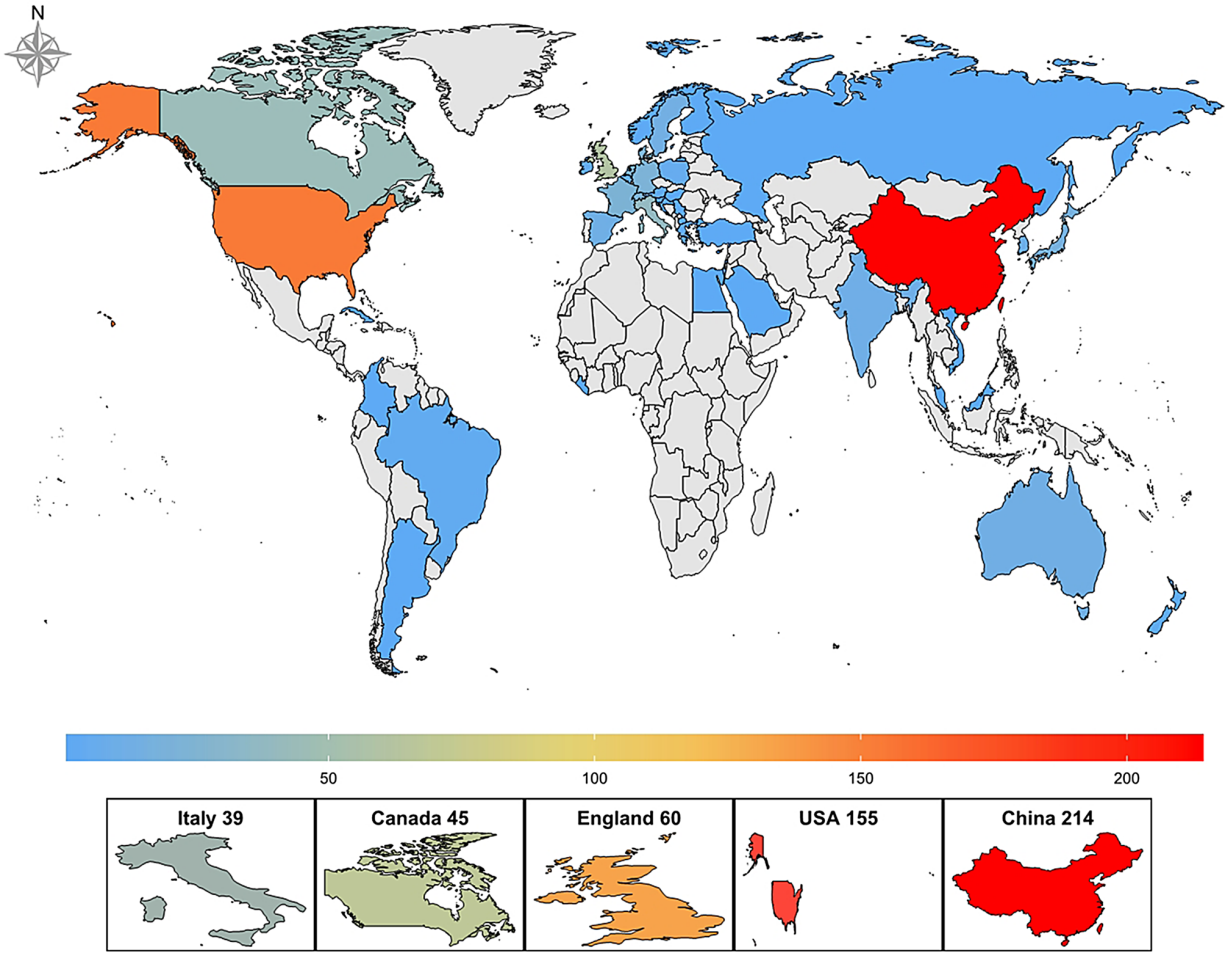
Geographical distribution of the countries/regions in amount of publications

**Fig. 4:**
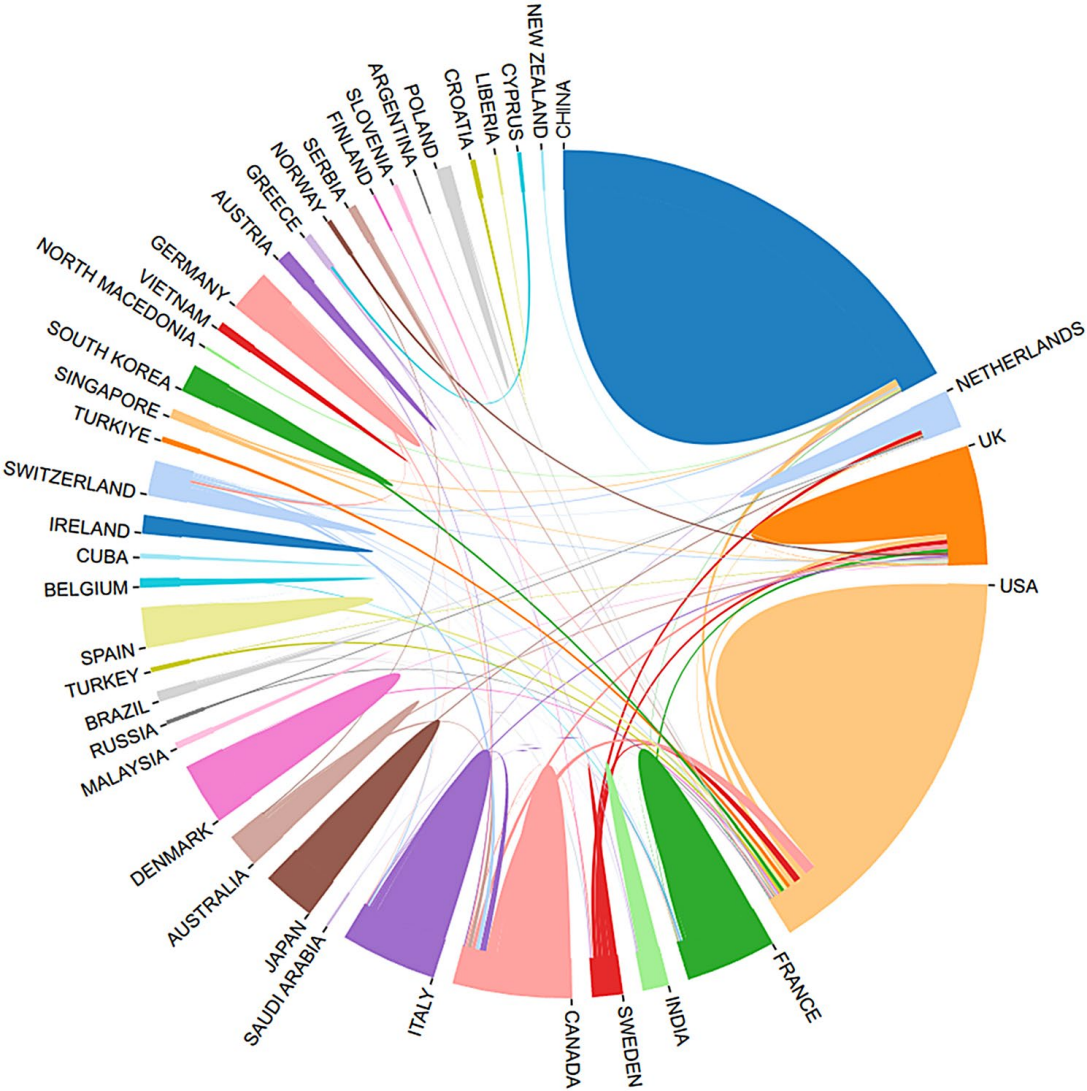
International collaboration among countries/regions. The co-authorship network diagram of (**A**) the countries/regions and (**B**) institutions. The size of the nodes represents the number of publications, the lines represent cooperation, and the color of the nodes represents the time

**Fig. 5:**
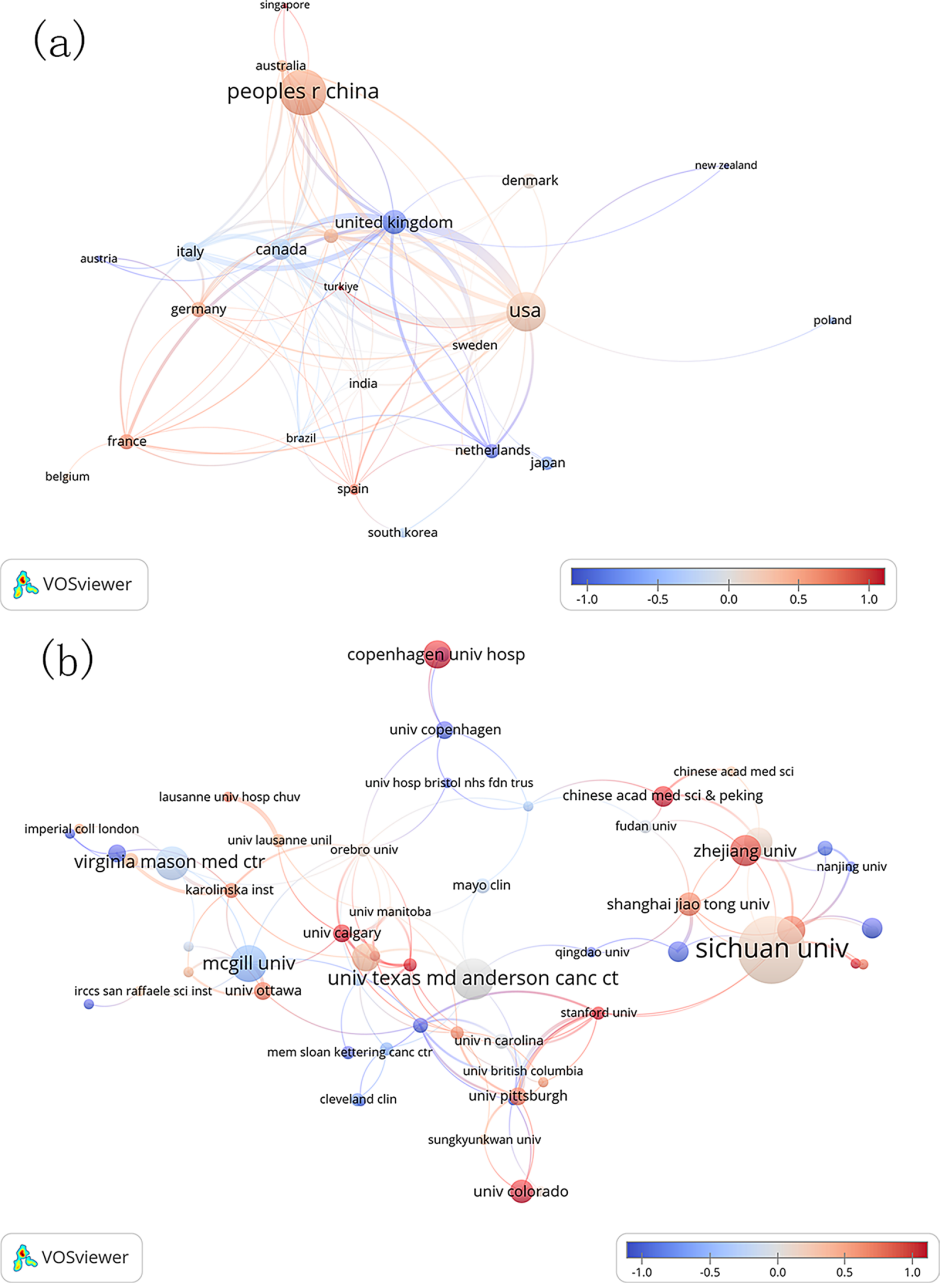
Overlays of co-authorship analysis and average publication time by countries/regions (**A**) and institutions (**B**). The lines represent cooperation, and the color of the nodes represents time

A total of 617 thoracic surgery-related ERAS papers were published across 44 countries/regions and 921 institutions. Figure 3 illustrates the geographical distribution of publications across countries/regions. The top three countries/regions by publication volume are China (214), the USA (155), and England (60). Detailed information on the top 10 countries/regions is provided in Table 1. Figure 4 depicts the international collaboration among countries/regions. The institutions with the highest number of publications are Sichuan University (26), The University of Texas MD Anderson Cancer Center (16), and McGill University (14). Table 1 also provides additional details on the top 10 institutions. Figure 5A and B present overlays of co-authorship analysis and average publication times by countries/regions and institutions, respectively. As shown in Fig. 5A, the USA has the most international collaborations, while the majority of the most recent papers on ERAS-related thoracic surgery research originate from France and Germany. As shown in Fig. 5B, most collaborations between institutions with a high number of publications are confined to the countries/regions in which they are based, with much of the latest research conducted at Copenhagen University Hospital and Zhejiang University.

**Table 1:** Top 10 productive countries/regions and top 10 productive institutions related to the ERAS in thoracic surgery

Rank	Country/Region	Count (%)	Citations	Rank	Institution	Count	Citations	Location
1	China	214 (34.68)	2245	1	Sichuan University	26	394	China
2	USA	155 (25.12)	4777	2	The University of Texas MD Anderson Cancer Center	16	692	USA
3	England	60 (9.72)	3561	3	McGill University	14	737	Canada
4	Canada	45 (7.29)	2571	4	Virginia Mason Medical Center	13	573	USA
5	Italy	39 (6.32)	1047	5	Zhejiang University	12	43	China
6	Denmark	24 (3.89)	1096	6	Nanjing medical University	11	134	China
7	Germany	23 (3.73)	481	7	Zhengzhou University	11	64	China
8	France	23 (3.73)	298	8	Harvard Medical School	11	340	USA
9	Switzerland	22 (3.57)	2020	9	Copenhagen university hospital	11	94	Denmark
10	Netherlands	21 (3.40)	984	10	Shanghai Jiao Tong University	9	36	China

### Analysis of journals

**Fig. 6:**
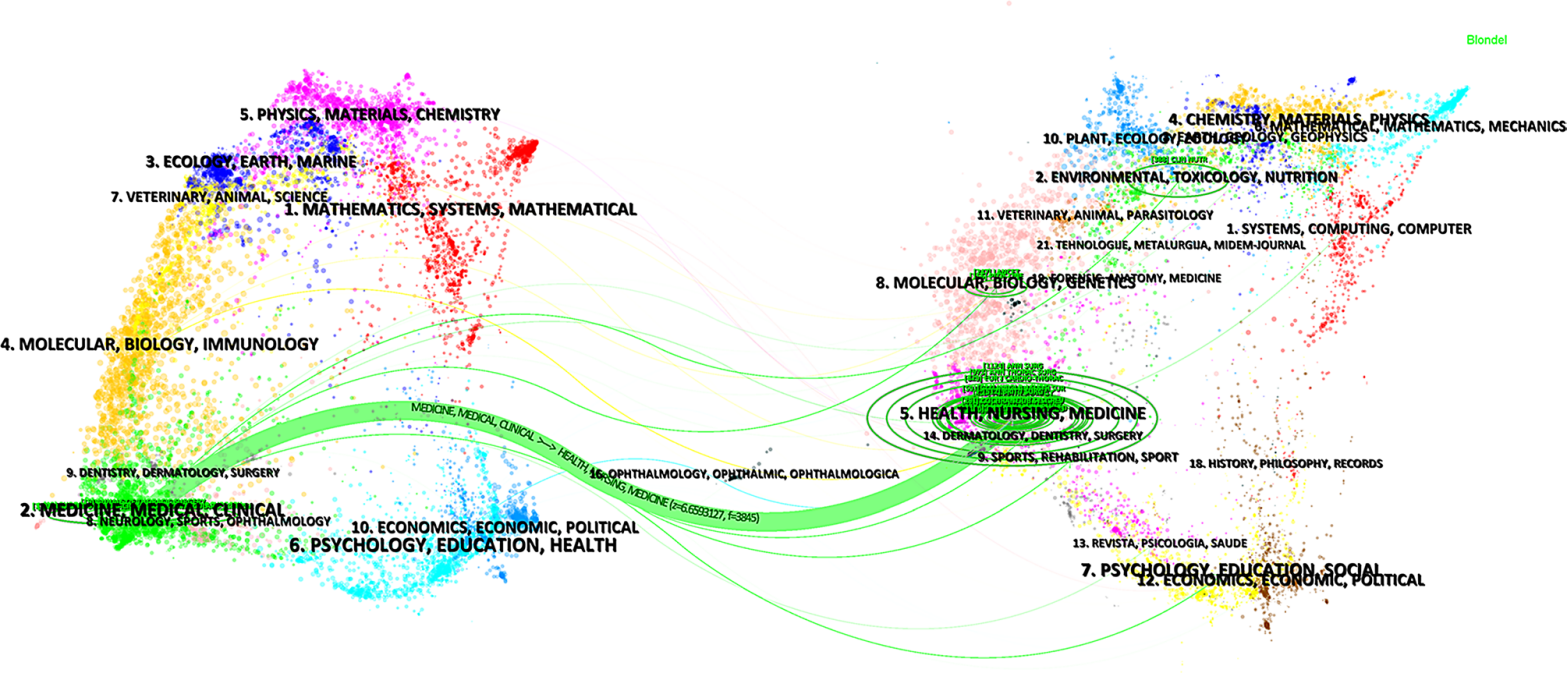
A dual-map overlap of journals of ERAS in thoracic surgery. The left is the cited journal and the right is the cited journal, the lines between cited journal and cited journal represent the citation relationship

A total of 617 papers were published across 222 journals. The journal with the highest number of publications was *Journal of Thoracic Disease* (60), followed by *Medicine* (22) and *Annals of Thoracic Surgery* (19). *Journal of Thoracic Disease* (1000), *European Journal of Cardio-Thoracic Surgery* (966), and *World Journal of Surgery* (752) are the top three journals in terms of citations. The majority (70%) of the top 10 journals by publication volume and citation are based in the USA. Table 2 provides a list of the top 10 journals by publication volume and citation. Figure 6 presents a dual-map overlap of journals, indicating that current ERAS-related thoracic surgery studies are predominantly published in journals focusing on Medicine/Medical/Clinical subjects, with a substantial proportion based on research from journals within the Health/Nursing/Medicine disciplines.

**Table 2:** Top 10 productive journals and top 10 highly cited journals related to the ERAS in thoracic surgery

Journal	Count (%)	Country	Journal	Citations	Country
Journal of Thoracic Disease	60 (9.72)	China	Journal of Thoracic Disease	1000	China
Medicine	22 (3.57)	USA	European Journal of Cardio-Thoracic Surgery	966	Netherlands
Annals of Thoracic Surgery	19 (3.08)	USA	World Journal of Surgery	752	USA
Diseases of the Esophagus	17 (2.76)	USA	Annals of Thoracic Surgery	565	USA
Journal of Clinical Medicine	13 (2.11)	Switzerland	Journal of Thoracic and Cardiovascular Surgery	548	USA
European Journal of Cardio-Thoracic Surgery	12 (1.94)	Netherlands	Annals of Surgery	360	USA
Journal of Thoracic and Cardiovascular Surgery	11 (1.78)	USA	Diseases of the Esophagus	354	USA
Journal of Cardiothoracic and Vascular Anesthesia	11 (1.78)	USA	Current Opinion in Anesthesiology	249	USA
World Journal of Surgery	9 (1.46)	USA	International Journal of Surgery	198	USA
Current Opinion in Anesthesiology	9 (1.46)	USA	British Journal of Anaesthesia	196	England

### Analysis of authors and co-cited authors

**Fig. 7:**
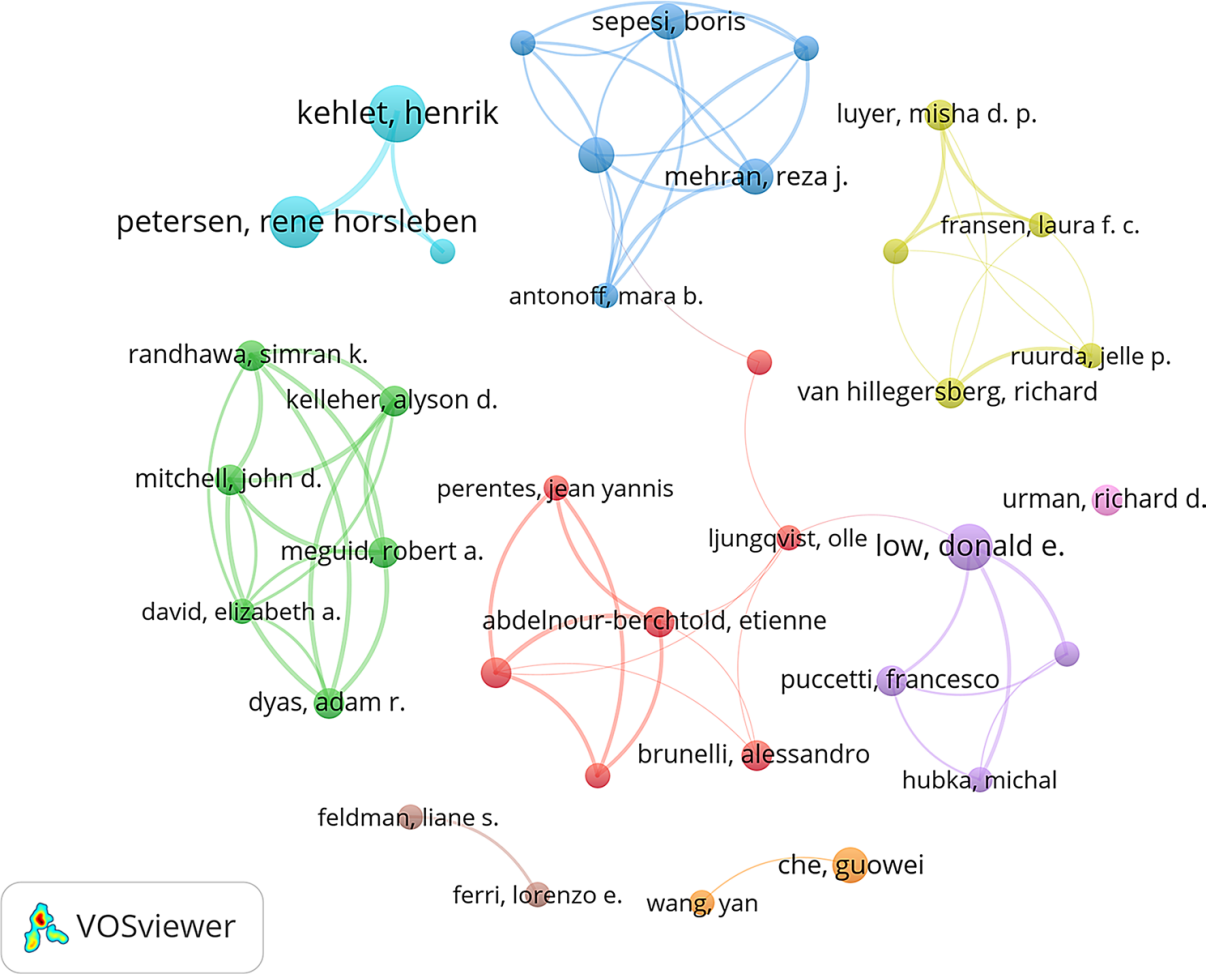
The co-authorship network map for the author

**Fig. 8:**
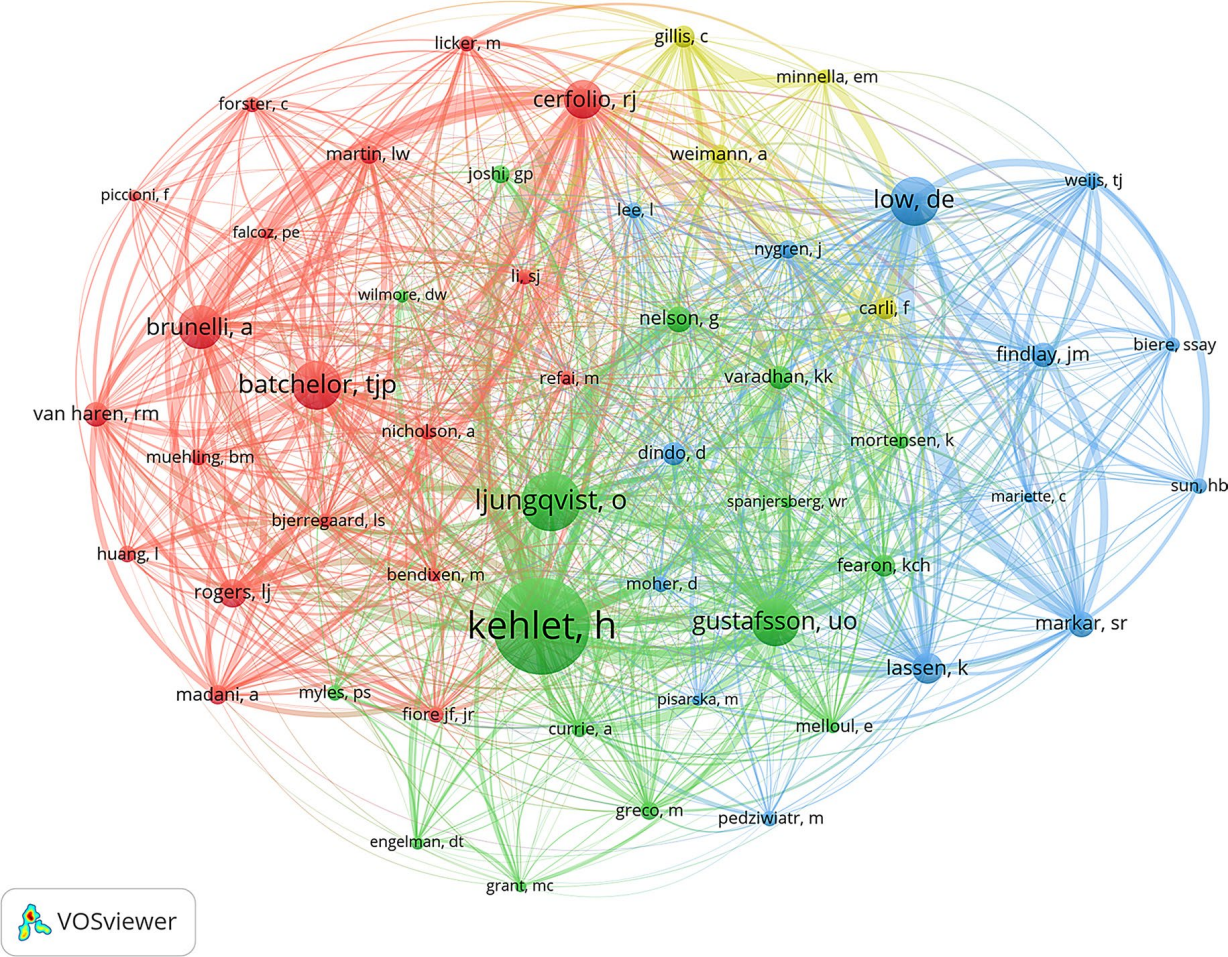
The co-citation network map of co-cited authors. The lines represent citations

A total of 617 ERAS research papers in thoracic surgery involved 3,397 authors. Table 3 presents the top 10 authors by publication volume and the top 10 co-cited authors by citation count. The top five authors by publication volume are Henrik Kehlet (11), Rene Horsleben Petersen (10), Donald E. Low (9), Reza J. Mehran (7), and Boris Sepesi (7). The top five co-cited authors by citation count are Henrik Kehlet (287), Olle Ljungqvist (163), Timothy J. P. Batchelor (133), Donald E. Low (130), and U. O. Gustafsson (129). In ERAS research on thoracic surgery, Henrik Kehlet, Donald E. Low, and Alessandro Brunelli are among the top 10 authors both in terms of publications and citations. These authors are also highly productive and influential. Figure 7 presents the co-authorship network of these authors. Figure 8 presents a co-citation network diagram, demonstrating that authors such as Henrik Kehlet exert significant influence.

**Table 3:** Top 10 productive authors and top 10 co-cited authors related to the ERAS in thoracic surgery

Rank	Authors	Documents	Rank	Co-cited Author	Citation
1	Henrik Kehlet	11	1	Henrik Kehlet	287
2	Rene Horsleben Petersen	10	2	Olle Ljungqvist	163
3	Donald E Low	9	3	Timothy J P Batchelor	133
4	Reza J Mehran	7	4	Donald E Low	130
5	Boris Sepesi	7	5	U O Gustafsson	129
6	Gabriel E Mena	7	6	Alessandro Brunelli	116
7	Guowei Che	7	7	Robert J Cerfolio	100
8	Francesco Puccetti	6	8	Kara Lassen	76
9	Richard van Hillegersberg	6	9	Luke J Rogers	71
10	Alessandro Brunelli	6	10	Gregg Nelson	63

### Analysis of references

**Fig. 9:**
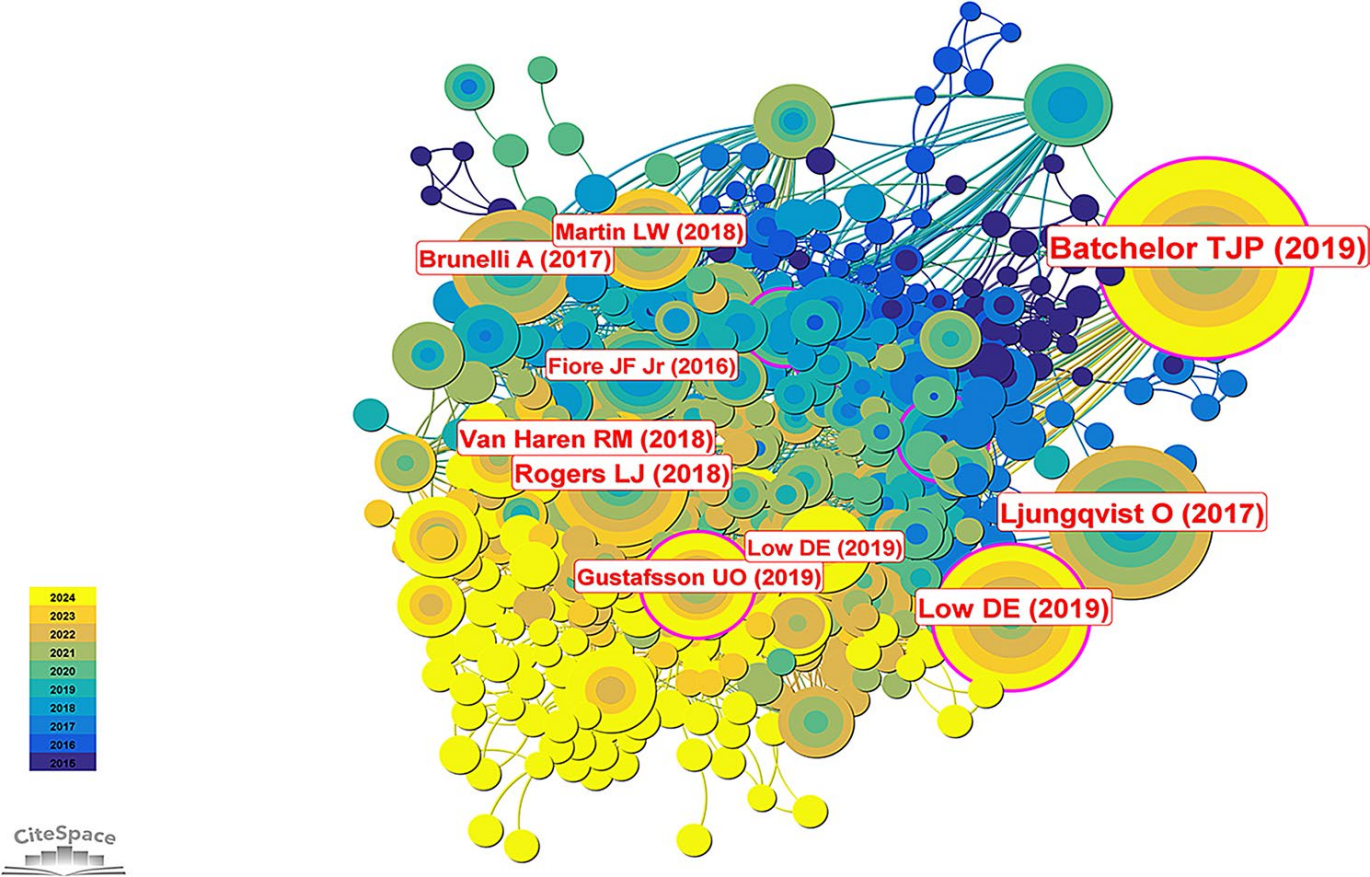
The co-cited network map of references. The purple rings on the outside of the nodes represent centrality

The included papers cited a total of 17,123 references. The top three references, in order of citations, were authored by Timothy J. P. Batchelor et al. [5] (111 citations), Olle Ljungqvist et al. [12] (68 citations), and Donald E. Low et al. [13] (60 citations). According to centrality, the top three references were authored by U. O. Gustafsson et al. [14] (0.13), Timothy J. P. Batchelor et al. [5] (0.12), and Donald E. Low et al. [13] (0.10). Table 4 presents details of the top 10 cited references. Figure 9 presents a co-citation network diagram of references, where centrality greater than 0.1 (indicated by purple rings around the nodes) signifies key node references. This demonstrates that 30% of the top ten references by citation are highly influential.

**Table 4:** Top 10 high-cited references related to the ERAS in thoracic surgery

Rank	References	First Authors	Citation	Centrality	Journal
1	Guidelines for enhanced recovery after lung surgery: recommendations of the Enhanced Recovery After Surgery (ERAS^®^) Society and the European Society of Thoracic Surgeons (ESTS)	Timothy J P Batchelor	111	0.12	European Journal of Cardio-thoracic Surgery
2	Enhanced Recovery After Surgery: A Review	Olle Ljungqvist	68	0.06	JAMA Surgery
3	Guidelines for Perioperative Care in Esophagectomy: Enhanced Recovery After Surgery (ERAS^®^) Society Recommendations	Donald E Low	60	0.10	World Journal of Surgery
4	The impact of enhanced recovery after surgery (ERAS) protocol compliance on morbidity from resection for primary lung cancer [18]	Luke J Rogers	50	0.04	Journal of Thoracic and Cardiovascular Surgery
5	Enhanced Recovery Decreases Pulmonary and Cardiac Complications After Thoracotomy for Lung Cancer [19]	Robert M Van Haren	44	0.02	Annals of Thoracic Surgery
6	Enhanced recovery pathway versus standard care in patients undergoing video-assisted thoracoscopic lobectomy	Alessandro Brunelli	39	0.02	Journal of Thoracic and Cardiovascular Surgery
7	Guidelines for Perioperative Care in Elective Colorectal Surgery: Enhanced Recovery After Surgery (ERAS^®^) Society Recommendations: 2018	U O Gustafsson	33	0.13	World Journal of Surgery
8	Implementing a Thoracic Enhanced Recovery Program: Lessons Learned in the First Year [17]	Linda W Martin	31	0.05	Annals of Thoracic Surgery
9	Systematic review of the influence of enhanced recovery pathways in elective lung resection [20]	Julio F Fiore Jr	25	0.03	Journal of Thoracic and Cardiovascular Surgery
10	Benchmarking Complications Associated with Esophagectomy	Donald E Low	24	0.05	Annals of Surgery

### Analysis of keywords

**Fig. 10:**
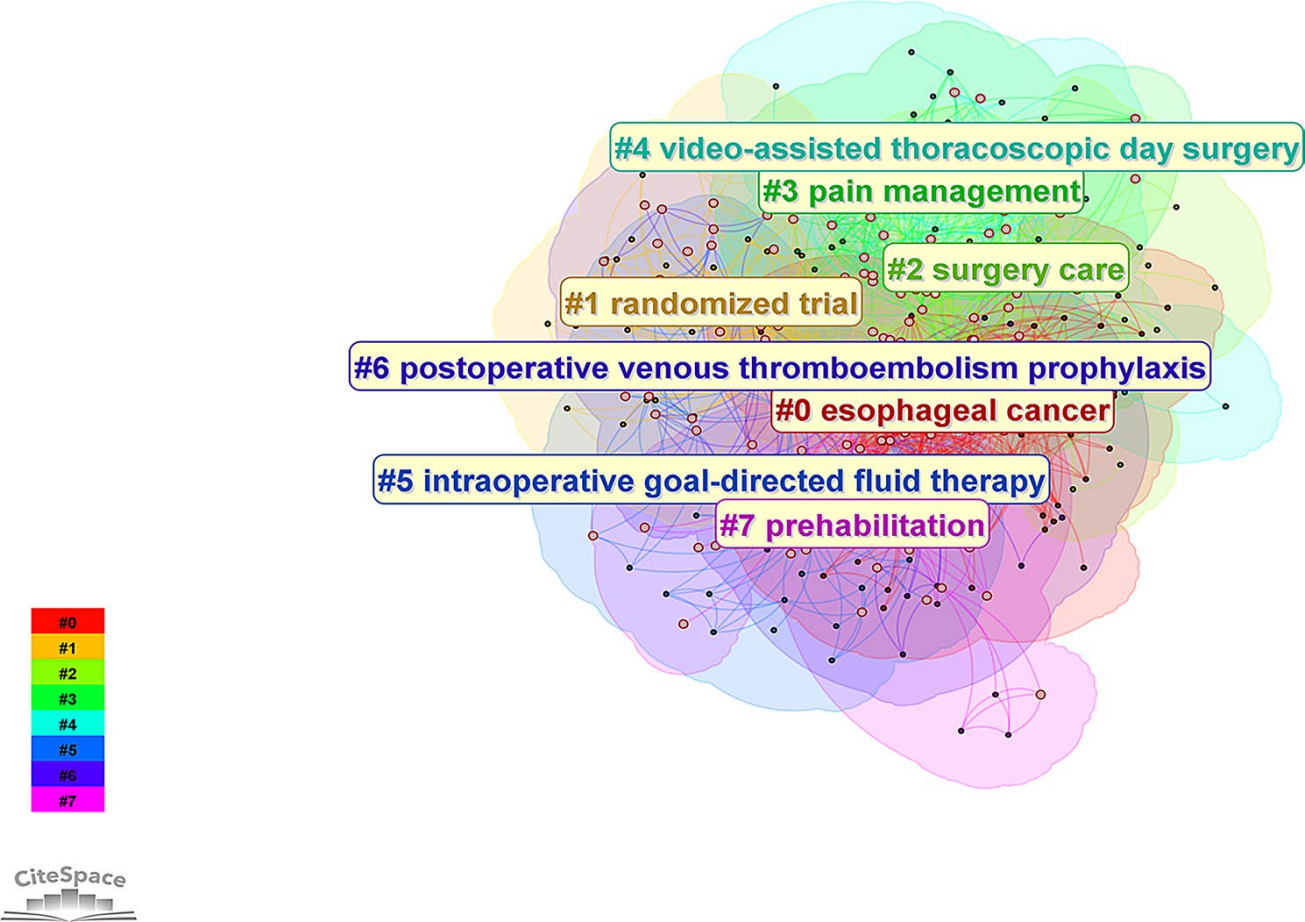
The cluster and co-occurrence map of keywords

**Fig. 11:**
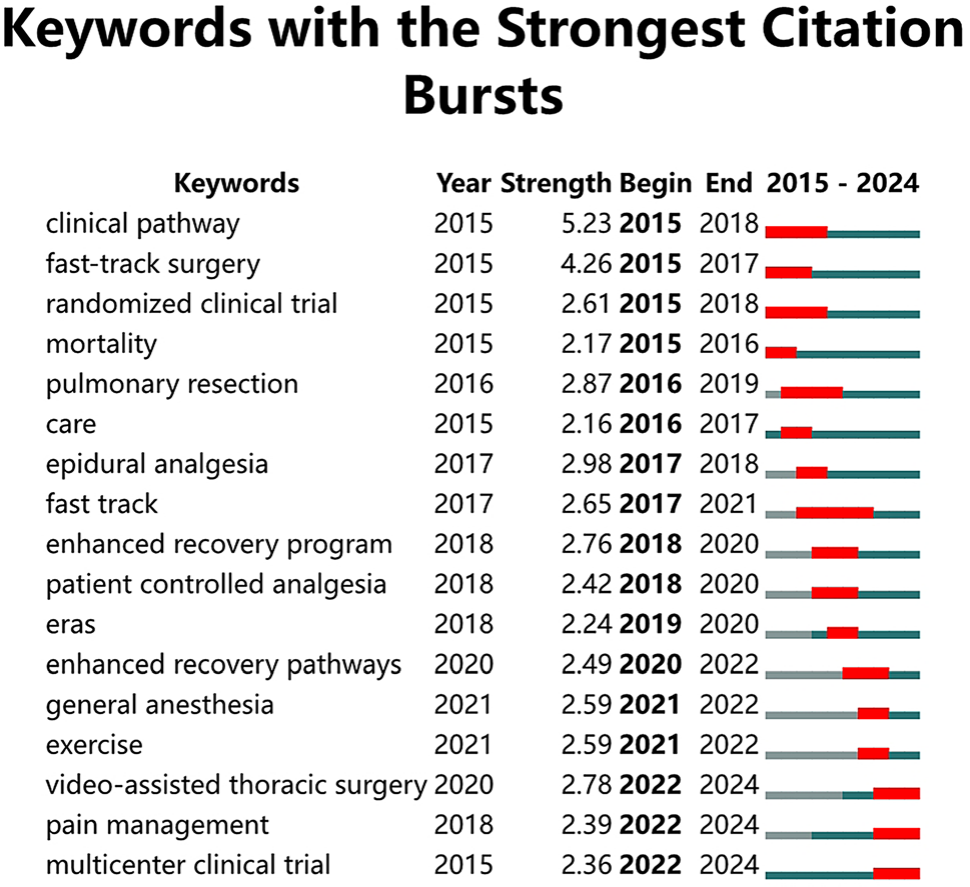
The burst detection map of keywords from the decade of ERAS in thoracic surgery

A total of 1,192 keywords were identified in the included papers. Figure 10 presents an overlay plot of the co-occurrence and cluster analysis of keywords. Eight keyword clusters were identified by cluster analysis and categorized into various topics, including: surgery-related (#4 video-assisted thoracoscopic day surgery); postoperative complications (#6 postoperative venous thromboembolism prophylaxis); nursing-related (#2 surgery care); anesthesia-related (#3 pain management, #5 intraoperative goal-directed fluid therapy); and other topics (#1 randomized trial, #0 esophageal cancer, #7 prehabilitation). Figure 11 presents a burst detection map of keywords from the past decade. The burst detection map highlights early research hotspots such as “fast-track surgery” and “clinical pathway,” with “clinical pathway” having the strongest burst strength at 5.23. Currently, the hottest keywords are “video-assisted thoracic surgery,” “pain management,” and “multicenter clinical trial.” These three keywords have remained highly relevant for an extended period and have been a prominent research topic since 2022.

## Discussion

Thoracic surgery involves operations on all chest organs, excluding the cardiovascular system, the majority of which are major and complex. Thoracic surgery requires the ERAS concept, as perioperative management is a multifaceted process, and enhancing surgical skills alone is insufficient to expedite patient recovery and ensure both physical and mental well-being. Although recent research in thoracic surgery has widely applied the ERAS concept, the overall development, current status, and future trajectory of the field remain unclear. This study addresses gaps in the development, current status, and future direction of the field through a bibliometric analysis of the countries/regions, institutions, journals, authors, references, and keywords from 617 ERAS papers published in thoracic surgery over the past decade.

The largest output of ERAS research in thoracic surgery comes from China (214 publications), followed by the USA (155 publications). China is also home to 50% of the top ten institutions with the highest number of publications. The USA and England are key contributors to international cooperation, as shown in Figs. 4 and 5A. However, China’s limited international collaboration is inconsistent with its high research output, highlighting the need for enhanced global cooperation. Institutions with high publication volumes exhibit some collaboration (Fig. 5B), but such cooperation is often confined to the institution’s country, which hampers scientific progress. In the future, increased collaboration between institutions across nations, as well as within countries, will be essential for advancing the field.

The *Journal of Thoracic Disease* is the leading journal, publishing nearly 10% of all ERAS-related papers in thoracic surgery, with a total of 60 publications. It also has the highest citation count, while the *European Journal of Cardio-Thoracic Surgery* ranks second. Despite publishing only 12 ERAS-related papers, this journal’s combined publication and citation impact is substantial, likely due to its publication of the ERAS guidelines. As shown in Table 2, the top 10 journals by publication volume and citations include the *European Journal of Cardio-Thoracic Surgery*, *Journal of Thoracic Disease*, *World Journal of Surgery*, *Annals of Thoracic Surgery*, *Current Opinion in Anesthesiology*, *Diseases of the Esophagus*, and *Journal of Thoracic and Cardiovascular Surgery*. These seven journals not only publish a substantial volume of ERAS-related thoracic surgery research but also exert considerable influence, indicating that research published in these journals is of high quality. The USA is home to the majority of the top 10 journals by publication volume and citations, underscoring its dominant position in the field. The dual-map overlap of journals (Fig. 6) reveals that fields such as Health/Nursing/Medicine form the foundation for current ERAS research, reflecting the overarching philosophy of ERAS. Health/Nursing fields are also closely linked to the success of ERAS. The volume of publications and citations reflects an author’s contribution and influence within the field.

Henrik Kehlet, Donald E. Low, and Alessandro Brunelli are recognized as leading experts in thoracic surgery-related ERAS research, ranking among the top ten academics globally for their contributions and influence. Henrik Kehlet, often referred to as the “father of ERAS,” is credited with first proposing the ERAS concept. Donald E. Low, one of the most prolific authors in the field, published the Guidelines for Perioperative Care in Esophagectomy: ERAS Society Recommendations [13] and the study Benchmarking Complications Associated with Esophagectomy [15], both of which are among the top 10 cited references. Alessandro Brunelli, the sixth-most cited author, has a highly influential paper titled “Enhanced Recovery Pathway versus Standard Care in Patients Undergoing Video-Assisted Thoracoscopic Lobectomy“ [16], which ranks among the top 10 most cited references. As shown in Table 3, these authors have made significant contributions to ERAS research in thoracic surgery, reflecting their substantial influence in the field.

The references reflect the primary focus of the field, with the top ten most cited references being published in surgery-related journals. The most cited reference, published in the European Journal of Cardio-Thoracic Surgery, is a guideline for implementing ERAS in lung surgery. Since lung surgery is the most common procedure in thoracic surgery, ERAS implementation in this context has attracted the most attention. Among the top 10 cited references are ERAS-related reviews, guidelines for perioperative care in thoracic surgery, and studies on the role of ERAS in lung surgery and esophagectomy, as well as efforts to reduce postoperative complications. Almost all of these references, regardless of their specific content or journal, are focused on surgical practices. While the ERAS concept is fundamentally based on surgery, it encompasses a comprehensive approach to rapid recovery throughout the perioperative period. Future research should aim to explore non-surgical aspects of ERAS, including nursing and anesthesia, with an emphasis on higher-quality studies.

The keyword clustering can be categorized into several groups, including surgery, postoperative complications, nursing, and anesthesia, as previously mentioned. As shown in Fig. 11, the rise in surgery-related keywords began early and has remained persistent, while bursts in terms like ‘care’ and ‘surgery care’ highlight the crucial role of perioperative care in implementing the ERAS concept in thoracic surgery. Esophageal cancer is another prominent keyword cluster, with esophagectomy being the primary treatment [21]. Enteral nutrition has become a key focus in ERAS treatment for esophageal cancer in recent years [22]. Early enteral nutrition following esophagectomy, compared to parenteral nutrition, has been shown to reduce pulmonary complications and improve nutritional support in patients with normal gastrointestinal function [23]. Moreover, providing appropriate enteral nutrition before esophageal surgery can accelerate gastrointestinal function recovery and reduce complications [24].

One of the current frontier topics is anesthesia. Thoracic surgery is almost always performed under general anesthesia, and anesthesia plays a key role in the ERAS protocol for thoracic surgery. In thoracic surgery, ERAS protocols for anesthesia are extensively used to prevent intraoperative hypothermia through body temperature control [25] and to reduce airway injury through proper airway management during single-lung ventilation [26, 27]. “Intraoperative goal-directed fluid therapy” is a key term in the anesthesia-related cluster. The use of fluid strategies like goal-directed fluid therapy enables precise fluid volume management, thus reducing postoperative complications. Fluid management is an essential aspect of ERAS anesthesia [28–30]. Another common keyword in the anesthesia-related cluster is ‘pain management,’ as patients undergoing thoracic surgery often experience significant postoperative pain. Techniques such as intercostal nerve block analgesia, paravertebral blockade, and epidural anesthesia are used to manage postoperative pain, thereby reducing the need for opioids [31, 32]. Reduced postoperative pain, assistance with sputum discharge, appropriate exercise, shortened recovery time, and prevention of chronic pain all contribute to improved long-term quality of life for patients. These benefits align closely with the principles of the ERAS philosophy.

Current research is focusing on ‘prehabilitation,’ as physical frailty has been linked to higher postoperative complications and lower long-term survival rates. It is recommended to engage in moderate exercise prior to surgery to increase physical reserves and prepare for the stress response induced by the procedure [33–35]. Furthermore, a key component of ERAS implementation in thoracic surgery is the effective organization of postoperative rehabilitation exercises.

One of the key steps in implementing ERAS in thoracic surgery is minimally invasive surgery, as evidenced by the growing adoption of video-assisted thoracic surgery (VATS), which remains a current hotspot. The integration of thoracoscopic technology and more advanced minimally invasive tools has significantly reduced the trauma associated with pulmonary surgery compared to traditional open surgery. These procedures not only shorten operation times but also reduce the risks associated with prolonged anesthesia, accelerating patient recovery [36]. VATS is also employed to remove mediastinal mass lesions, offering benefits such as fewer complications, shorter hospital stays, and more cosmetically appealing incisions [37].

In contrast, open transthoracic esophagectomy for esophageal cancer involves greater surgical trauma. Minimally invasive esophagectomy, utilizing thoracoscopy and laparoscopy, offers advantages including shorter operation times, less intraoperative bleeding, shorter hospital stays, and fewer postoperative complications compared to open transthoracic esophagectomy [38]. Patients undergoing minimally invasive esophagectomy experience less trauma and recover more rapidly. Moreover, these patients exhibit better 5-year survival rates compared to those who undergo open transthoracic esophagectomy [39].

The addition of robotic-assisted surgery further enhances the advantages of minimally invasive surgery. Robotic-assisted surgery offers reduced trauma and a more streamlined procedure compared to thoracoscopic and laparoscopic surgery due to the flexibility of robotic arms and superior visual clarity. Complex esophageal, lung, and mediastinal surgeries are increasingly performed with robotic assistance [40–43]. The primary objective of these advanced techniques, including VATS, minimally invasive esophagectomy (MIE), and robotic-assisted surgery, is to ensure effective oncological outcomes. It is also critical to tailor the treatment approach based on the patient’s individual circumstances.

Notably, “video-assisted thoracoscopic day surgery” is one of the emerging keywords in ERAS research. As ERAS continues to evolve in thoracic surgery, day surgery [44] has become feasible for some patients undergoing VATS, a development made possible by the collaborative efforts of multidisciplinary teams. This trend is expected to expand, allowing more thoracic surgeries to be performed as day procedures, maximizing the benefits of ERAS and enabling faster patient recovery.

Another significant research focus is the “multicenter clinical trial,” which plays a crucial role in improving clinical outcomes within ERAS protocols for thoracic surgery. High-quality, multicenter randomized clinical trials are essential for providing evidence-based support for ERAS practices in thoracic surgery. It is anticipated that well-designed trials will continue to drive the application of ERAS in this field.

While these keywords highlight the current hotspots in thoracic surgery-related ERAS research, the essence of ERAS lies in multidisciplinary collaboration across the entire perioperative period. ERAS incorporates education, preoperative assessment, smoking cessation, and alcohol restriction, among other elements. Other key components include early preoperative antibiotic administration, short preoperative fasting periods [45], avoidance of indwelling catheters for patients with short surgical durations and normal voiding function, prevention of postoperative blood clots, and the strategic use of drainage tubes. Postoperative wound care and timely complication management, including early removal of gastrointestinal decompression tubes and drainage tubes, are all integral parts of the ERAS philosophy.

Thoracic surgery-related ERAS research has made significant progress and has been increasingly integrated into clinical practice. However, there are still challenges to overcome in the application of ERAS in thoracic surgery. Despite widespread adoption in many thoracic surgery centers, the quality of outcomes has been inconsistent. This variability underscores the need for strict adherence to the ERAS protocol in clinical settings across all institutions. Furthermore, the successful implementation of ERAS requires a multidisciplinary approach, engaging not only thoracic surgeons but also nursing staff, oncologists, imaging specialists, respiratory therapists, anesthesiologists, and laboratory professionals. There is an urgent need to deepen current research, expand its scope, and broaden the field of ERAS in thoracic surgery. Although much of the current literature focuses on thoracic surgery, anesthesiology, and nursing, the depth of research remains insufficient, and many studies lack high-level evidence. Indeed, the predominance of retrospective and single-center studies in the existing ERAS literature limits the reliability of the available evidence. To address this limitation, future research should focus on well-designed multicenter randomized controlled trials (RCTs) with adequate sample sizes, standardized ERAS protocols, and long-term follow-up outcomes. In addition, prospective cohort studies incorporating cost-effectiveness analyses, patient-reported outcomes, and real-world implementation barriers would further strengthen the evidence base. These methodological improvements will help overcome current research limitations and facilitate the development of widely applicable clinical guidelines for thoracic ERAS.

## Limitation

There are several limitations to this study. First, although the Web of Science Core Collection offers extensive coverage of peer-reviewed scientific literature and is one of the most widely used databases in bibliometric research, even with its comprehensive bibliometric indicators, full record data, and citation references—which are essential for conducting rigorous bibliometric analyses—the database has inherent limitations. The standardized metadata format of WoS facilitates cross-study comparisons, and limiting the data source to WoS ensures that the study can be replicated by other researchers using a widely recognized and accessible platform. Furthermore, this approach helps avoid potential variability introduced by overlapping or inconsistent indexing across multiple databases. Nonetheless, we acknowledge that the exclusion of other major databases such as PubMed, Scopus, and Embase may have resulted in the omission of certain relevant publications. Differences in indexing strategies and journal coverage across databases may have affected the overall comprehensiveness of the dataset. Secondly, this study excluded non-English publications and non-article/review document types. While this decision was made to ensure optimal compatibility with bibliometric tools such as VOSviewer and CiteSpace, and to enhance the scientific rigor and comparability of the results, it must be acknowledged that relevant insights may exist in non-English literature or gray literature sources (e.g., editorials, conference abstracts). Nevertheless, these materials were excluded to maintain methodological consistency and to focus exclusively on peer-reviewed and validated scientific contributions. Additionally, some recent high-impact publications may not have accumulated many citations due to the time constraints of the study, although we believe this does not significantly affect the overall conclusions.

## Conclusion

In conclusion, this study utilizes bibliometric analysis to examine and discuss the evolution of ERAS in the field of thoracic surgery, as well as its current research landscape, hotspots, and future research trends. ERAS-related research in thoracic surgery has maintained a high volume in recent years, with significant potential for future growth. ERAS is a comprehensive concept that spans the entire perioperative period, and numerous studies have demonstrated its benefits in thoracic surgery, particularly in minimally invasive surgery, anesthesia, and nursing care. This study elucidates the global research trends, key contributing institutions, and emerging hotspots in ERAS within the field of thoracic surgery. By identifying underexplored areas and visualizing international collaboration networks, our findings may serve as a valuable reference for clinical researchers and healthcare policymakers in guiding resource allocation, prioritizing future research directions, and fostering multicenter collaborations. Moreover, recognizing the leading countries and institutions can facilitate the dissemination and standardization of ERAS protocols, ultimately promoting evidence-based implementation in clinical practice. However, research in other areas remains insufficient, and further studies are needed to focus on additional aspects of the perioperative period and to delve deeper into current research directions. The hotspots and future research trends of the ERAS concept in thoracic surgery include innovations in minimally invasive techniques, prudent selection of anesthesia methods, and analgesic strategies. The successful application of the ERAS pathway in thoracic surgery relies on the support of high-level evidence-based medical data, yet much of the existing ERAS research in related fields is limited by retrospective designs and single-center studies. To strengthen the evidence base, future studies should prioritize well-designed multicenter RCTs with adequate sample sizes, standardized ERAS protocols, and extended follow-up periods. Additionally, prospective cohort studies that incorporate cost-effectiveness analyses, patient-reported outcomes, and real-world implementation barriers may further strengthen the evidence base. These methodological enhancements are essential for overcoming current limitations and advancing the development of widely applicable, evidence-based clinical guidelines for thoracic ERAS.

## Data Availability

The original data used in this study are available in the Web of Science database. (https://www.webofscience.com).
